# Knowledge of and attitudes towards abortion among adolescents in Lao PDR

**DOI:** 10.1080/16549716.2020.1791413

**Published:** 2020-08-03

**Authors:** Viengnakhone Vongxay, Kongmany Chaleunvong, Dirk R. Essink, Jo Durham, Vanphanom Sychareun

**Affiliations:** a Faculty of Public Health, University of Health Sciences, Vientiane, Lao PDR; b Institute Research and Education Development, University of Health Sciences, Vientiane, Lao PDR; c Faculty of Earth and Life Sciences, Athena Institute for Research on Innovation and Communication in Health and Life Sciences, Vientiane, Lao PDR; d School of Public Health and Social Work, Queensland University of Technology, Brisbane, Australia

**Keywords:** LEARN: Sexual Reproductive Health, ANC and Nutrition, Adolescence, abortion, knowledge, attitude, Lao PDR

## Abstract

**Background:**

Adolescents are at high risk of unintended pregnancy and consequent unsafe abortion. Evidence from Lao PDR suggests a high but underreported prevalence of induced abortion, especially amongst adolescents. Research suggests adolescents are less likely to have an unsafe abortion when they have accurate knowledge about abortion and hold positive attitudes towards abortion.

**Objective:**

The purpose of this study was to investigate awareness and attitudes towards abortion and associated factors in Lao PDR.

**Methods:**

This study used a descriptive, cross-sectional design. The study was conducted between January and May 2019 in two different provinces within Lao PDR, namely, Khammouane and Champasack provinces. Participants included in- and out-of-school male and female adolescents (n = 800). Data were collected using a structured questionnaire and entered into the EPI Data version 3.1. All analysis was undertaken using STATA v.13. Univariate analysis and frequency distributions were used to study the pattern of responses and bivariate descriptive analysis to report attitudes and knowledge by participant characteristics. The association between participant characteristics and overall scores of attitudes towards abortion was evaluated using multiple logistic regression.

**Findings:**

Most respondents (78.8%) were aware of the processes and potential consequences of becoming pregnant at a young age. One-third of respondents (31.5%), were aware of induced abortion. Of those, only 12.1% held positive attitudes towards induced abortion. Factors associated with positive attitudes towards abortion were ethnicity, mother’s education and ever having had sex.

**Conclusion:**

In the case of unintended or unwanted pregnancy, adolescents must also have adequate knowledge and access to safe abortion and associated counselling services. This study suggests a need to increase sexual and reproductive health literacy including information about safe abortion. This requires a holistic approach to sexual education and needs the support and involvement of adolescents themselves as well as parents, community members and healthcare workers.

## Background

Globally, abortion is a leading cause of maternal mortality and morbidity. It is estimated that 7–9% (95% CI 4.7–13.2) of all maternal deaths are due to spontaneous or induced abortion, with most of these maternal deaths occurring in low and middle-income countries [1]. Induced abortion is often a result of lack of an unmet need for contraceptives [2] and where abortion laws are restrictive, unintended abortion may be resolved in circumstances where abortion is unsafe, illegal or both [1–3]. The risk of dying from an unsafe abortion is particularly high and complications can include genital trauma or a foreign body in the uterus, vagina or cervix, and sepsis or peritonitis [4,5]. Long-term complications from non-fatal, unsafe induced abortion can include ectopic pregnancy, chronic reproductive tract and pelvic infection and infertility [4,6]. Economic costs of unsafe abortion include the direct costs of providing medical care for women hospitalised due to complications of unsafe abortion and indirect costs related to loss of productivity from abortion-related morbidity and mortality [4].

The most effective way to prevent unintended pregnancy is to use a modern contraceptive method. For many sexually active adolescents, however, and especially those who are unmarried, and living in low and middle-income countries, access to contraception can be challenging [3,7,8]. Barriers to access include affordability, being able to research a service point and social norms around adolescent sex [3,7–9]. Even when adolescents have access to contraceptives, they may find it difficult to achieve consistent and correct use, dislike available methods, be unable to negotiate safe sex or choose a contraceptive method that suits them [3,7,8]. Non-use or failure of contraceptive methods places adolescent females at risk of unintended pregnancy and unsafe abortion [1–3,7,10]. Even where abortion is legal, for adolescents it may be unaffordable, practitioners may be reluctant to perform abortions, or adolescents may not know about, or be able to access safe abortion [9]. Adolescents in low socio-economic groups and living in a community without access to an appropriate sexual and reproductive health facility can be particularly vulnerable to the negative outcomes of unsafe abortion [3–5,10].

Understanding adolescent knowledge of, and attitudes towards, abortion can shed light on some of the needs of adolescents and help develop interventions. While scant, some research has examined adolescents’ knowledge and attitudes towards abortion. Factors associated with attitudes towards abortion include religious affiliation, religious attendance, educational experiences, circumstances of the pregnancy and political affiliation [11–13]. Studies have generally found no association between gender and attitudes towards abortion [11,12]. Some research suggests an association between age and but the evidence is mixed [11,12]. Mothers’ and friends’ attitudes have also been associated with individual attitudes towards abortion [14]. Studies have also found students with sexual experience are more likely to hold positive attitudes towards abortion than their counterparts with no sexual experience [11,12,14]. Some research, while not conclusive, indicates a positive relationship between knowledge, either through knowing someone who has had an abortion or through formal education [11,13–16] and adolescents’ decision-making in relation to abortion [14,17].

Lao PDR is a lower-middle-income country in South East Asia. The adolescent fertility rate at 65 per 1000 adolescents aged 15–19 years is the highest in the region [18] and given the limited access adolescents have to family planning, it is likely that at least some of these pregnancies are unintended. Data on abortion are difficult to obtain, however, as until recently abortion has been governed by the Criminal Code Article 92 (1990) and available only if legally approved, e.g. to save mothers from pregnancy-related complications [19]. Where data are available, they suggest widespread prevalence of unsafe abortion. A hospital-based descriptive study reported 40% of presentations due to complications following induced abortions were among 20–24 year olds. Most patients had used what is called locally, the ‘Chinese drug’ (a combination of anti-progestin and prostaglandin), readily purchasable from pharmacists [20]. The Young Women’s Sexual Behaviour study conducted in Vientiane among respondents aged 15–24 years, also provides evidence of unintended pregnancies being resolved through abortion with 23.2% of participants reporting having engaged in vaginal sex also having had an abortion [21]. Most of the participants who had undergone an abortion were young and unmarried and used medication (61.2%) [21]. In 2016, the first Maternal Death Review (MDR) indicated 54% of maternal deaths were due to post-partum haemorrhage, many of which the Ministry of Health attributed to unsafe abortion.

Following the MDR, Guidelines to Prevent Unsafe Abortion were developed and are now considered the legal framework and clinical standard for safe abortion services [22,23]. These guidelines also provide a more liberalised regimen, including pre-abortion counselling [22,23]. There is limited understanding, however, of adolescents’ knowledge and attitudes towards abortion. This is an important gap given the high rate of adolescent fertility, low levels of sexual and reproductive health literacy, and that many adolescents do not complete upper secondary school, which is the only level where sexual education is provided [24]. This study begins to fill this gap and importantly, unlike most studies investigating adolescents’ attitudes towards abortion and associated factors includes younger adolescents (11–14 years). The study is particularly timely as the new Guidelines to Prevent Unsafe Abortion are being rolled out and as access to safe abortion becomes more accessible.

## Methods

This study used a descriptive, cross-sectional design and was conducted between January and May 2019. The study included in- and out-of-school male and female adolescents (n = 800). The sample size was determined by estimating the proportion of positive attitude toward abortion as 50% and margin of error 5%; plus 30% estimated possible drop-out or incompletion due to using a self-administered questionnaire. The calculated sample size was 785 subjects, which was rounded up to 800 for implementation. The study was undertaken in Khammouane province in the central part of the country and Champassak province in the south. These two provinces were selected based on convenience and because most sexual health studies with adolescent participants have previously been undertaken in the northern parts of the country [8,25–27]. For both provinces, available reports do not disaggregate data related to induced abortion by age (other than 15–49 years). No data is available related to pregnancy or induced abortion is available for adolescents below the age of 15 years.

Khammuane Province consists of ten districts and according to the most recent census, has a population of 392,052 of which 22.0% are aged 10–19 years [28]. The adolescent birth rate is estimated to be 71 per 1000 adolescents (aged 15–19 years) and the proportion of induced abortion among women aged 15–49 years was 4.6% with an abortion rate 0.2% [18].

Champasack Province is the most southerly province of Lao PDR and also consists of ten districts. The population is 694,023 of which 21.3% are adolescents aged 10–19 years [28]. Adolescent pregnancy is estimated to be 50 per 1000 adolescents aged 15–19 years and the proportion of induced abortion among women aged 15–49 years was 3.7% with an abortion rate of 0.1% [18].

Participants were aged 11–19 years of age. To select potential participants, first, simple-random sampling was applied by constructing a list of all high schools in the province and then randomly selecting three schools. In each selected school, a list of students in each grade for the academic year 2018–2019, from primary grade 5 to upper and lower high school grade 1–7 was prepared (each selected high school also prepared the list of primary grade 5 students from its associated primary school). Simple random sampling was then used to identify potential participants. The sample size for each school was determined using probability proportional to size [29].

For out-of-school adolescents, two districts were selected using simple random sampling. Following this, a list of villages in each of the selected districts was prepared and simple random sampling applied to select five villages in each district. In each selected village, a list of out-of-school adolescents was prepared with the assistance of the village head and potential participants selected using simple random sampling. The number of selected females and males was determined based on probability proportional to size of each selected village [29].

After obtaining permission from school directors, the purpose of the study was explained to the students in the classroom by the researchers. Written consent was obtained from all in-school adolescents aged 15–19 years. With ethical approval, participants aged 15–17 years old, also gave consent without guardian consent, based on the assumption young people of this age group are competent (Gillick Competency principle). This is consistent with Lao family law which recognises young people aged 15–17 are able to provide informed consent. For all younger participants, the study was explained to the adolescent and their guardian and informed consent sought. Thereafter, a researcher also explained the purpose of the study to the adolescent in private to confirm consent. For out-of-school adolescents, with ethical approval written or verbal informed consent was obtained depending on the literacy of the participant. All participants were invited to ask the research assistants questions related to the study.

In-school participants completed the questionnaires themselves in class while, due to low levels of literacy, face-to-face interviews with trained researchers asking the questions were used for out-of-school adolescents. Both in-and out-of-school participants could ask the researchers questions if they did not understand the wording of any questions.

The questionnaire consisted of five parts, namely socio-demographic characteristics of respondents (age, grade, ethnicity, religion, level of education, classroom, parent’s educational level, occupation and self-reported socio-economic status); source of information on pregnancy and consequences of pregnancy; knowledge of abortion, attitudes towards abortion; ever having had sex, abortion practices and parent-adolescent sexual communication. Questions related to abortion were only answered by those participants who stated they had heard of abortion. The other sections, however, were answered by all participants.

Questions related to knowledge of abortion comprised 13 items (e.g., whether to have an abortion should be a woman’s personal decision; abortion is allowed under selected circumstances; medication that can be used for abortion). Where participants answered the question of knowing of medication or substance for induced abortion, the answer of Misoprostol/Cytotec was recorded as correct. Questions related to knowledge of abortion were coded ‘correct’ or ‘incorrect’ and summed up to provide a composite score which was then categorised as a ‘high’ or ‘low’ level of knowledge. The cut-off-point for a high knowledge level was equal to, or higher than the mean, of the total score. Where the total score was less than the mean, it was coded a low level of knowledge.

There were six items relating to attitudes towards abortion (e.g. abortion is a sin; women should have access to safe abortion services) with items having both positive and negative statements. A four-point Likert scale ranging from ‘“strongly agree”’ to ‘“strongly disagree”’ was used to assess responses. Response codes were as follows: 1 = strongly agree, 2 = agree, 3 = disagree and 4 = strongly disagree. Items 1, 2 and 6 were reversed when coded for statistical analyses (1 = strongly disagree, 2 = disagree, 3 = agree and 4 = strongly agree). Participants’ positive attitudes towards abortion were summed up to provide a composite score with a possible total score ranging from 12 to 60. The median attitude towards abortion score was 15, which was used as a cut-off.

There were nine items related to abortion practices (e.g., where do unmarried women in this community go to have an abortion?; Where do friends or females in this community go to have an abortion?; What methods can be used to terminate a pregnancy or start your period, etc.). Questions on parent-sexual communication with adolescents were also included and asked about the difficulty or ease with which participants could talk to their parents about important things, including sexual heath and safe sex.

### Statistical analysis

The data was entered into the EPI Data version 3.1 for cleaning and then the statistical software STATA v.13 for analysis. Univariate analysis and frequency distributions were used to study the pattern of responses and bivariate descriptive analysis to report attitudes and knowledge by participant characteristics. The association between participant characteristics and the overall score of attitudes towards abortion was evaluated using multiple logistic regression. Crude and adjusted odds ratios with 95% confidence intervals were calculated, with odds ratios excluding unity constituting statistical significance (p < 0.05).

### Ethical approval

The University of Health Sciences Institutional Review Board (IRB) approved the protocol for this study. Consent was obtained from all adolescents aged 18–19 years and, with ethical approval, participants aged 15–17 years old gave consent based on the assumption young people of this age group are competent (Gillick Competency principle). For younger adolescents, aged 11–15 years, informed assent was obtained from the young person as well as from their guardians/parents. For confidentiality, names were not collected on the form.

## Findings

### Socio-demographic characteristics of adolescents

Eight hundred adolescents, comprising 433 females and 367 males formed the study sample. The majority of the respondents (52.5%) were between the ages of 11–15 years with a minimum age of 11 years and maximum of 19 years; with a mean age of 14.9 and standard deviation of 2.25. In total, 94.9% were Lao-Tai ethnic and 92.8% identified as being Buddhist. Three-quarters were currently school-going. Around 14.6% of participants said their father’s education level was up to upper secondary school, but for mothers this was only 9.9%. The majority of adolescents (92.8%) rated their family socio-economic status as moderate (see Table 1 for more socio-demographic information).

**Table 1.** Socio-demographic characteristics of 800 adolescent males and females in Lao PDR.

	Males		Females		Total	
Characteristics	*n* = 367	%	*n* = 433	%	*n* = 800	%
Age (Mean 14.9, SD 2.2)						
10–15	176	48.0	244	56.4	420	52.5
16–19	173	47.1	163	37.6	336	42.0
No answer	18	4.9	26	6.0	44	5.5
Ethnicity						
Lao-Tai	336	91.6	423	97.7	759	94.9
Mon-Khmer	24	6.5	9	2.1	33	4.1
No answer	7	1.9	1	0.2	8	1.0
Education						
Primary or less	53	14.4	53	12.2	106	13.3
Lower-upper secondary	307	83.7	375	86.6	682	85.3
College or higher	7	1.9	5	1.2	12	1.5
School-going						
Yes	269	73.3	331	76.4	600	75.0
No	98	26.7	102	23.6	200	25.0
Current class/grade						
Primary grade 5	38	14.1	52	15.7	90	15.0
Lower secondary (Grade 1–4)	134	49.8	178	53.8	312	52.0
Lower secondary (Grade 5–7)	83	30.9	81	24.5	164	27.3
Other/none	14	5.2	20	6.0	34	5.7
Fathers’ highest level of school						
Primary or less	61	16.6	82	18.9	143	17.8
Lower-upper secondary	105	28.6	122	28.1	227	28.4
College or higher	162	44.1	199	46.0	361	45.1
Other/unknown	39	10.6	30	6.9	69	8.6
Father having a job						
Yes	256	78.0	331	82.1	587	80.3
No	67	20.4	61	15.1	128	17.5
Other/unknown	5	1.5	11	2.7	16	2.2
Mothers’ highest schooling						
Primary or less	117	33.3	140	33.3	257	33.3
Lower-upper secondary	91	25.9	124	29.5	215	27.9
College or higher	143	40.7	156	37.1	299	38.8
Other/unknown						
Mother having a job?						
Yes	257	73.2	304	72.4	561	72.8
No	90	25.6	110	26.2	200	25.9
Other/unknown	4	1.1	6	1.4	10	1.3
Perceived socio-economic status						
Rich	6	1.6	2	0.5	8	1.0
High Middle	12	3.3	7	1.6	19	2.4
Middle	312	85.0	402	92.8	714	89.3
Poor	31	8.4	18	4.2	49	6.1
Very poor	0	0.0	1	0.2	1	0.1
Other/unknown	6	1.6	3	0.7	9	1.1

### Source of information on pregnancy and consequences of pregnancy among adolescent

Table 2 shows that the majority of respondents (78.8%) were aware of the processes of becoming pregnant and its consequences. Regarding preferred sources of sexual and reproductive health information participants said school teachers (24.6%), followed by social media (17%) and TV/radio (15.7%). Participants wanted more information on planning for pregnancy (57.9%), signs of pregnancy (45.9%), and unplanned/unwanted pregnancy (35.7%). More females than males were interested in these topics as follows: planning for pregnancy (62.3% vs 52.4%), signs of pregnancy (49.9% vs 40.7%) and abortion methods (20.3% vs 14.5%). In total, 66.7% of participants had attended classes in school on pregnancy-related topics, with females attending more than males (69.9% vs 62.5%). Slightly more than half of the participants (59%), said there should be more sexuality classes, with more females than males stating this (56.4% vs 62.1%).

### Parent-adolescent sexual communication

Table 3 illustrates reported parent-adolescent sexual communication. Females typically preferred to speak to mothers rather than fathers about sexual matters (36.5% vs 18.4%) and found it more difficult to discuss important things with fathers than mothers (18.9% vs 9.1%). Males also reported finding it easier to discuss sexuality with mothers than fathers (40.2% vs 30.2%) although females said they were more likely to discuss sexual matters with their mothers compared to males (48.6% vs 21.7%).

**Table 2.** Source of information on pregnancy and consequences of pregnancy information among 800 adolescent males and females in Lao PDR.

Information source on pregnancy	Males		Females		Total	
	*n* = 367	%	*n* = 433	%	*n* = 800	%
Adolescents heard about pregnancy and consequences?						
Yes	275	74.9	355	82.0	630	78.8
No	85	23.2	73	16.9	158	19.8
No answer	7	1.9	5	1.2	12	1.5
Sources adolescents preferred?						
Printed or broadcast media	45	20.2	68	10.7	113	13.2
Social media	26	11.7	81	12.8	107	12.5
School or teacher	72	32.3	109	17.2	181	21.1
Peers/Friends/Project	36	16.1	59	9.3	95	11.1
Mother/Father/Brother/other	44	19.7	317	50.0	361	42.1
Topics where adolescents would like to know more						
(Multiple answers allowed)						
How to get pregnant	48	17.5	49	13.8	97	15.4
Planning for pregnancy	144	52.4	221	62.3	365	57.9
Unplanned/unwanted pregnancy	87	31.6	138	38.9	225	35.7
Signs of pregnancy	112	40.7	177	49.9	289	45.9
Pregnancy testing	22	8.0	47	13.2	69	11.0
Abortion methods	40	14.5	72	20.3	112	17.8
Attending school classes on pregnancy and consequences of pregnancy						
Yes	172	62.5	248	69.9	420	66.7
No	45	16.4	44	12.4	89	14.1
Don’t know	58	21.1	63	17.7	121	19.2
The need of (more) learning on pregnancy, consequences of pregnancy and right (n-420)						
More about pregnancy & consequences	97	56.4	154	62.1	251	59.8
Less about pregnancy & consequences	7	4.1	8	3.2	15	3.6
More about right	67	39.0	83	33.5	150	35.7
No answer	1	0.6	3	1.2	4	1.0

**Table 3.** Parent-adolescent sexual communication among 800 adolescent males and females in Lao PDR.

Communications	Males		Females		Total	
	*n* = 367	%	*n* = 433	%	*n* = 800	%
Ease of talking with father						
Easy/very easy	145	44.2	105	26.1	250	34.2
Average	111	33.8	204	50.6	315	43.1
Difficult/very difficult	57	17.4	76	18.9	133	18.2
Do not see him	2	0.6	7	1.7	9	1.2
Other/unknown	13	4.0	11	2.7	24	3.3
Discussing sex-related matters with father						
Often	10	3.2	8	2.1	18	2.6
Occasionally	48	15.3	62	16.1	110	15.8
Never	254	81.2	310	80.5	564	80.8
Other/unknown	1	0.3	5	1.3	6	0.9
Talking to father about safe sex						
Never	279	76.0	350	80.8	629	78.6
Once	14	3.8	10	2.3	24	3.0
A few times	49	13.4	44	10.2	93	11.6
Often	23	6.3	21	4.8	44	5.5
Other/unknown	2	0.5	8	1.8	10	1.3
Ease of talking with mother						
Easy/very easy	207	59.0	240	57.1	447	58.0
Average	102	29.1	139	33.1	241	31.3
Difficult/very difficult	33	9.4	38	9.0	71	9.2
Do not see her	3	0.9	1	0.2	4	0.5
Other/unknown	6	1.7	2	0.5	8	1.0
Discussing sex-related matters with mother						
Often	15	4.4	41	9.8	56	7.4
Occasionally	59	17.3	162	38.8	221	29.1
Never	265	77.5	212	50.8	477	62.8
Other/unknown	3	0.9	2	0.5	5	0.7
Talking to mother about safe sex						
Never	258	70.3	283	65.4	541	67.6
Once	21	5.7	24	5.5	45	5.6
A few times	50	13.6	77	17.8	127	15.9
Often	28	7.6	43	9.9	71	8.9
Other/unknown	10	2.7	6	1.4	16	2.0

### Knowledge of abortion

One-third of respondents (31.5%) were aware of induced abortion. Of those who had heard of induced abortion (71%) believed the decision on whether to have an abortion should be the female’s personal choice. Most of these participants (78.6%) agreed a person should have an abortion where to continue the pregnancy would endanger a woman’s life, or in the case of rape (62.3%); where there was a fetal abnormality (74.6%); the women was single (57.9%) or to continue her study (62.7%).

Among adolescents who had heard of abortion, the mean score on the knowledge about abortion index was 5.2 ± 3.8 based on a scale from 0–10, with 10 the highest possible score. Of participants who had heard of abortion, 47.6% had a high level of knowledge, with females having a higher knowledge scores than males (53.2% vs. 38.5%, respectively) (see Table 5).

**Table 4.** Knowledge of abortion among 800 adolescent males and females in Lao PDR.

Questions	Males		Females		Total	
	*n* = 367	%	*n* = 433	%	*n* = 800	%
Heard about abortion?						
Yes	96	26.2	156	36.0	252	31.5
No	257	70.0	260	60.0	517	64.6
Other/unknown	14	3.8	17	3.9	31	3.9
Abortion should be a woman’s personal decision*						
Yes	62	64.6	117	75.0	179	71.0
No	34	35.4	29	18.6	63	25.0
No answer	0	0.0	10	6.4	10	4.0
Abortion is allowed in the condition*						
If the Pregnancy Endangers woman’s Life	73	76.0	125	80.1	198	78.6
If the child might be born deformed	66	68.8	122	78.2	188	74.6
If pregnancy resulted from rape	59	61.5	98	62.8	157	62.3
If Family cannot afford to support the child	61	63.5	112	71.8	173	68.7
If the woman is not married	58	60.4	88	56.4	146	57.9
If a young female or woman wants to continue her studies	49	51.0	89	57.1	138	54.8
Knowing of any medication or substance a woman can take if she wants to have an abortion*						
Yes	35	10.6	19	18.6	54	12.5
No	33	10.0	2	2.0	35	8.1
Don’t know	241	72.8	80	78.4	321	74.1
Medication or substance do you know*						
Misoprostol/Cytotec*	4	11.4	0	0.0	4	10.8
Chloroquine	1	2.9	1	50.0	2	5.4
Boiled roots	11	31.4	0	0.0	11	29.7
Painkillers/antibiotics	6	17.1	0	0.0	6	16.2
Beverages	10	28.6	0	0.0	10	27.0
Physical removal	3	8.6	0	0.0	3	8.1
Crushed bottles (drink ground glass)	1	2.9	0	0.0	1	2.7
Washing powder (Dynamo, Boom, etc.)	3	8.6	0	0.0	3	8.1
Unspecified tablets	5	14.3	0	0.0	5	13.5
Tablets inserted vaginally	16	45.7	0	0.0	16	43.2
Does not know	1	2.9	0	0.0	1	2.7

*The questions 2, 3.1 to 3.7, 4, 5 were summed up as composite score.

Cronbach’s alpha = 0.65 (reasonable).

**Table 5.** Knowledge and attitudes towards abortion among 800 adolescent males and females in Lao PDR.

Knowledge and Attitude	Level	Males	Females	Total
		*n*^c^ (%)	*n*^c^ (%)	*n*^c^ (%)
Knowledge^a^ *[Mean: 5.2 (±3.8); Min-max: 0–10]*	Higher Knowledge	37 (38.5)	83 (53.2)	120 (47.6)
	Lower knowledge	59 (61.5)	73 (46.8)	132 (52.4)
Attitude^b^	Positive attitude towards abortion	23 (6.7)	29 (7.2)	52 (7.0)
	Negative attitude towards abortion	319 (93.3)	371 (92.8)	690 (93.0)

^a^Score of cut-off-point for the high knowledge level is equal or higher than mean of the total score. Mean knowledge score of males = 5.0(±1.9); min-max: 0–10. Mean knowledge score of females = 5.4(±2.0); 0–10.

^b^The cut-off-point of attitude score is ≥ 80%, indicating positive attitude and the cut-off-point attitudinal score less than 80% denoted negative attitude towards abortion. The attitude raw score of min-max is 6–24.

^c^Number of observations between knowledge and attitude was different. The total number of observations for knowledge was 252, including only those who had heard of abortion. The total number of observations for attitude was 742/800 due to some non-responses.

Only a few participants (12.5%) knew medical abortion or substances that could be taken to induce abortion, although females knew more than males (18.6% vs. 10.6%, respectively). Those aware of medical abortion methods cited tablets inserted vaginally (43.2%), boiled roots (29.7%), beverages (27%), painkillers/antibiotics (Cafenol, Panadol, ampicillin, aspirin, Anadin) (16.2%), Misoprostol/Cytotec (10.2%), and physical removal (8.1%).

### Attitudes towards abortion

Of the adolescents who had knowledge of abortion, most held negative attitudes towards abortion (93.0%), with little difference between males and females (see Table 5). Most respondents (71%) agreed or strongly agreed abortion is a sin, with females agreeing with this statement more than males (76.1% vs 65.2%). Additionally, 41.6% of respondents felt seeking an abortion was a sign of promiscuity in females, with males agreeing with statement more than females (43% vs 40.5%). In addition, 46.7% of these participants felt abortion was acceptable within the community where the gestational age was <3 months. Further, 68.3% agreed women should have access to safe abortion services, with females (71%) having a higher level of agreement than males (65.0%). More than half of respondents (62.1%) strongly agreed abortion can be fatal when performed in unsafe conditions. In addition, 44.6% of respondents agreed (and strongly agreed) it is not acceptable to talk about abortion, with no significant differences between males and females (see Table 6).

**Table 6.** Attitude towards abortion among 800 adolescent males and females in Lao PDR.

Statements reflecting attitude towards abortion	Males		Females		Total***		Total answers
	SD & D*	A & SA**	SD & D*	A & SA**	SD & D*	A & SA**	
	*n* (%)	*n* (%)	*n* (%)	*n* (%)	*n* (%)	*n* (%)	
Woman seeking abortion as promiscuity (*n* = 764)	199 (57.0)	150 (43.0)	247 (59.5)	168 (40.5)	446 (58.4)	318 (41.6)	764
Abortion as committing sin (*n* = 766)	121 (34.8)	227 (65.2)	100 (23.9)	318 (76.1)	221 (28.9)	545 (71.0)	766
Community’s belief on abortion acceptable if the GA is < 3 months (*n* = 747)	199 (57.7)	146 (42.3)	199 (49.5)	203 (50.5)	398 (53.3)	349 (46.7)	747
Women should have access to safe abortion services(*n* = 757)	121 (35.0)	225 (65.0)	119 (29.0)	292 (71.0)	240 (31.7)	517 (68.3)	757
A woman can die from an abortion done in unsafe conditions or by untrained providers (*n* = 755)	137 (39.8)	207 (60.2)	150 (36.5)	261 (63.5)	287 (38.0)	468 (62.0)	755
It is not acceptable to talk about any abortion-related issue (*n* = 751)	186 (54.1)	158 (45.9)	230 (56.5)	177 (43.5)	416 (55.4)	335 (44.6)	751

Cronbach’s alpha = 0.719; *Strongly disagree and disagree; **Agree and strongly agree.

***The number of answers was less than 800 because of non-responses to some statements.

### Factors associated with attitudes towards abortion

Table 7 shows the results of the multiple regression analysis and factors associated with attitudes towards abortion. The factors associated with positive attitudes towards abortion were ethnicity (crude OR: 2.3; p < 0.03; borderline significance in the adjusted model); mothers with college-level education (adjusted OR: 3.3; p < 0.02); ever had sex (adjusted OR: 3.8; p < 0.01).

**Table 7.** Factors associated with positive attitudes towards abortion (among 586 of 800 adolescents who responded to all relevant questions) in Lao PDR.

Factors	Above/below median score on positive attitudes towards abortion						
	*n* (%)		Crude OR		Adjusted OR		
	>median	≤median	OR	95%CI	OR		95%CI
Age group							
10–15 years	160 (50.2)	159 (49.8)	(ref)		(ref)		
16–19 years	176 (65.9)	91 (34.1)	1.9*	1.4–2.7	0.8		0.4–1.8
Sex							
Male	138 (53.7)	119 (46.3)	(ref)		(ref)		
Female	198 (60.2)	131(39.8)	1.3	0.9–1.8	1.8		0.8–3.7
Ethnicity							
Lao	308 (56.1)	241 (43.9)	(ref)		(ref)		
Mon-Khmer	28 (75.7)	9 (24.2)	2.4*	1.1–5.3	2.7		0.9–7.9
Currently school-going							
Yes	243 (56.0)	191 (44.0)	(ref)		(ref)		
No	93 (61.2)	59 (38.8)	1.2	0.8–1.8	1.8		0.8–3.8
Father’s education							
Primary or less	74 (63.2)	43 (36.8)	(ref)		(ref)		
Secondary	108 (57.8)	79 (42.2)	0.8	0.5–1.3	1.0		0.4–2.9
College or more	154 (54.6)	128 (45.4)	0.7	0.4–1.1	0.8		0.3–2.4
Father working							
No	61 (57.6)	45 (42.4)	(ref)		(ref)		
Yes	275 (57.3)	205 (42.7)	1.0	0.6–1.5	1.1		0.4–3.2
Mother’s education							
Primary or less	122 (59.8)	82 (40.2)	(ref)		(ref)		
Secondary	95 (59.8)	64 (40.2)	1.0	0.7–1.5	1.0		0.3–3.2
College or more	119 (53.4)	104 (46.6)	0.8	0.5–1.1	3.3*		1.3–8.7
Mother working							
No	93 (60.4)	61 (39.6)	(ref)		(ref)		
Yes	243 (56.2)	189 (43.8)	0.8	0.6–1.2	1.0		0.4–2.5
Father-adolescent sexual communication							
Low	262 (58.3)	187 (41.7)	(ref)		(ref)		
High	74 (54.0)	63 (46.0)	0.8	0.6–1.2	1.2		0.4–3.3
Mother-adolescent sexual communication							
Low	216 (54.8)	178 (45.2)	(ref)		(ref)		
High	120 (62.5)	72 (37.5)	1.4	0.9–2.0	1.8		0.7–4.5
Parent-adolescent safe-sex discussion							
Low	246 (55.2)	200 (44.8)	(ref)		(ref)		
High	90 (64.3)	50 (35.7)	1.5	1.0–2.2	1.1		0.3–3.7
Heard about pregnancy and consequences							
No	93 (44.5)	116 (55.5)	(ref)		(ref)		
Yes	243 (64.5)	134 (35.5)	2.3*	1.6–3.2	1.4		0.7–3.0
Ever had sex							
No	312 (57.3)	232 (42.7)	(ref)		(ref)		
Yes	18 (57.1)	24 (42.9)	1.0	0.5–1.9	3.8*		1.4–10.3

*p < 0.05

## Interpretations

This is the first study to the authors’ knowledge examining adolescents’ awareness and attitudes towards abortion and associated factors in the Lao PDR. Positively, most participants felt abortion should be the decision of the woman and women should have access to safe abortion. Nevertheless, most participating adolescents held conservative attitudes towards abortion. An adolescent holding conservative attitudes towards abortion may experience an unplanned birth or resolve the pregnancy through unsafe abortion with potentially long-term health consequences [11,13–15,30]. Conservative attitudes may be due to the previously restricted abortion law and negative social constructions of pre-marital sex and abortion.

As in other lower-middle-income countries, we observed low levels of abortion-related knowledge [30,31]. Low levels of knowledge might partly be due to their age, because we included a wide age-range (10 years old-19 years old), levels of knowledge however did not increase with older age. While adolescents’ knowledge was low, as reported elsewhere, males were less knowledgeable about abortion than the females [30]. Low levels of knowledge may also be a contributing factor to the conservative attitudes. Some participants were aware of misoprostol, which is often available over the counter at pharmacies.

Adolescents had various trusted sources of information including teachers. Information, which may be readily available but may also be inaccurate, was also sourced from social media and television. Adolescents may not be able to discern accurate from inaccurate information or safe or unsafe practices [30]. Communication with parents and more highly educated mothers was positively associated with attitudes towards safe abortion and confirms the importance of parent-adolescent communication regarding sexuality, reproductive health and skills development [32–35]. The study also affirms adolescents with sexual experience tend to hold more positive attitudes towards abortion [11,16,30]. This is likely to be because some of those who were sexually experienced had undergone an abortion or had least thought about the possibility of unintended pregnancy and how this might be resolved [36,37].

Participants of Mon-Khmer ethnicity held more positive attitudes towards abortion, than their non-Mon-Khmer peers, although few Mon-Khmer subjects participated. The Mon-Khmer are one of the 49 official ethnic groups in Lao PDR and constitute around 22% of the total population. It is not clear why this might be, but most of the Mon-Khmer participants were out-of-school and living in rural areas where adolescents have earlier sexual debut and a higher fertility rate than urban areas [18,28]. Ethnic minority populations in Lao PDR also typically hold more liberal attitudes towards pre-marital sex. Most of the research on ethnic minorities and sexuality however have been in northern Lao PDR [8,25–27] and further research is warranted in central and southern provinces.

This study suggests a need to increase sexual and reproductive health literacy. Adolescents must have adequate knowledge about, and access to, modern contraceptives, feel empowered to choose contraceptive methods suitable for them and be able to negotiate safe sex with their partners [24]. Additionally, adolescents need access to accurate and developmentally appropriate information related to and safe abortion [2,3,30]. The new Guidelines to Prevent Unsafe Abortion should be widely disseminated to that adolescents wishing to terminate a pregnancy are aware of where and how they can access safe abortion [15,22,23,30]. Also important is ensuring that safe abortion is affordable for adolescents, that health workers are trained in the new guidelines and that healthcare workers treat adolescents seeking abortion with respect and compassion.

As most educational interventions in Lao PDR are targeted at older school going adolescents, community outreach educative and skills building programmes are also needed [38,39]. Comprehensive sexual and reproductive health education should also be included earlier in the school curricula. The importance of parents being involved in discussions with adolescents should not be understated [30,34]. Many parents however may find sexual conversations with adolescents uncomfortable and healthcare workers or other professionals may be able to help parents develop effective strategies for having sexual conversations with their adolescent children [34]. Also important is working with healthcare providers and community members to identify and reduce any barriers to adolescents accessing family planning services [40].

A limitation of this study is its cross-sectional design which means the study only snapshot of abortion-related knowledge and attitudes at a certain point in time. Additionally, the two provinces were the study was undertaken were selected based on a convenience sample and because most previous studies have focused on northern provinces [8,25–27]. This means, however, the study is not nationally representative. A strength of the study is it included both in- and out-of-school adolescents, which is important the school-attendance rate in Lao PDR is low, although the proportion of in and out of school adolescents was different to the national census [28]. Despite these limitations, the study provides insight into abortion-related knowledge and attitudes towards abortion perceived by adolescents, on a vulnerable but understudied group in Lao PDR in regards to family planning and abortion research. Furthermore, unlike most studies we included a wide age-range with the minimum age of respondents was 11 years old and the maximum age 20 years old. While our intent was to recruit participants up to an including 19 years of age, some participants were 20 years old as they were in one of the included school grades, usually at upper secondary level.

### Conclusion

This study suggests there is a generally a low knowledge of and negative attitudes towards safe abortion exist among adolescents in Lao PDR, a country where adolescent pregnancy is high. A holistic, multi-sector approach to sexual education to in-and out-of-school adolescents that meets their needs and supports involvement of adolescents as well as parents, community members and healthcare workers. A comprehensive, holistic approach could improve adolescent reproductive health indicators and increase adolescents’ autonomy in meeting their sexual health needs, having benefits for individuals, families, communities and broader society. Further research is also warranted to inform holistic, evidence-based practices, programmes and policies to reduce negative attitudes towards adolescent sex, unintended pregnancy and abortion to minimize the risk of adolescents unsafe abortion.

## Paper context

Evidence suggests the Lao PDR has a high, but underreported, prevalence of induced abortion, especially amongst adolescents. Adolescents are less likely to have an unsafe abortion when they have accurate abortion-related knowledge and positive attitudes towards abortion. This study investigated abortion-related awareness and attitudes and associated factors in Lao PDR.
